# Identification and Description of Novel Mood Profile Clusters

**DOI:** 10.3389/fpsyg.2017.01958

**Published:** 2017-11-21

**Authors:** Renée L. Parsons-Smith, Peter C. Terry, M. Anthony Machin

**Affiliations:** School of Psychology and Counselling, University of Southern Queensland, Toowoomba, QLD, Australia

**Keywords:** affect, BRUMS, emotion, mood profiling, online assessment

## Abstract

Mood profiling has been a popular assessment strategy since the 1970s, although little evidence exists of distinct mood profiles beyond the realm of sport and exercise. In the present study, we investigated clusters of mood profiles derived from the six subscales of the Brunel Mood Scale using the *In The Mood* website. Mood responses in three samples (*n* = 2,364, *n* = 2,303, *n* = 1,865) were analyzed using agglomerative, hierarchical cluster analysis, which distinguished six distinct and theoretically meaningful profiles. K-means clustering further refined the final parameter solution. Mood profiles identified were termed the iceberg, inverse iceberg, inverse Everest, shark fin, surface, and submerged profiles. Simultaneous multiple discriminant function analysis showed that cluster membership was correctly classified with a high degree of accuracy. Chi-squared tests indicated that the six mood profiles were unequally distributed according to the gender, age, and education of participants. Future research should investigate the antecedents, correlates and consequences of these six mood profile clusters.

## Introduction

Mood has been described as “a set of feelings, ephemeral in nature, varying in intensity and duration, and usually involving more than one emotion” (Lane and Terry, 2000, p. 17). Mood measurement has typically occurred using self-report scales to assess transient emotions. The individualized and subjective nature of moods and emotions mean that responses elicited from self-report measures are considered to provide valid and useful information (Paulhus and Vazire, 2007). The 65-item Profile of Mood States (POMS; McNair et al., 1971, 1992) and, more recently, the abbreviated 24-item Brunel Mood Scale (BRUMS; Terry et al., 1999, 2003) have been used extensively to assess mood responses across a range of diverse contexts.

The most popular applications of mood profiling have been in the sport and exercise domains. More specifically, the role of mood in predicting sport performance has generated a considerable body of knowledge (see Beedie et al., 2000). The mental health model (Morgan, 1985) predicts that psychological health, as reflected by positive mood, associates with athletic success, whereas psychopathology associates with an increased incidence of failure (Rowley et al., 1995). The graphical representation of mood responses, proposed by Morgan (1980) to be typical of successful athletes approximates the shape of an iceberg, where the mean scores of the normative group represent the water line beneath which most scores fall. Parsimoniously termed the *iceberg profile*, this pattern of mood responses combines high vigor with low tension, depression, anger, fatigue, and confusion scores. The iceberg profile has subsequently been found to be the typical profile reported among athletes, successful or otherwise, and therefore is less indicative of athletic success than previously claimed (Renger, 1993; Terry and Lane, 2000).

Two additional mood profiles have previously been identified in the literature. The first profile, referred to as the *Everest profile* (Terry, 1995), is a more pronounced iceberg profile, characterized by higher vigor scores (>60%) and lower tension, depression, anger, fatigue, and confusion scores (<40%), and associates with superior performance. The second profile, referred to as the *inverse iceberg profile*, is characterized by below average scores for vigor and above average scores for tension, depression, anger, fatigue, and confusion, and typically debilitates performance efforts (Terry, 1995). Research now suggests that athletic performance is closely related to mood for some individuals but relatively independent of mood for others (Totterdell, 1999; Lane and Chappell, 2001).

From an applied practitioner perspective, mood profiling has utility for predicting the performance of athletes by means of individualized assessment of idiosyncratic mood-performance relationships (Terry, 1995). Mood profiling has been used to monitor adaptations to rigorous training schedules (Prapavessis et al., 1992; Raglin and Morgan, 1994), assess risk of athlete staleness or burnout caused by overtraining (Morgan et al., 1987), and monitor recovery from overtraining syndrome (Terry, 2004), defined as an acute or chronic state of incompetence causing decreases or plateaux in performance ability (Lemyre et al., 2007). The inverse iceberg mood profile may provide an important diagnostic indicator for overtraining syndrome (Budgett, 1998) and may possibly be indicative of a range of mental health disorders.

Additional applications within the sport context include monitoring psychological responses to travel fatigue and jetlag (Terry and Lane, 2011), assessing the effectiveness of injury rehabilitation programs (Pearson and Jones, 1992) and quantifying the mood benefits of music (Terry et al., 2012). Mood profiling can also help to discriminate athletes at risk of eating disorders, with BRUMS scores successfully screening out athletes not at risk of pathogenic behaviors with 91% efficiency (Terry and Galambos, 2004).

Beyond the realm of sport, mood profiling has been used as a screening tool for post-traumatic stress risk in military populations. For example, van Wijk et al. (2013) found that a BRUMS cut-off score of ≥ 24 for total mood disturbance (i.e., sum of scores for tension, depression, anger, fatigue and confusion minus vigor score) at demobilization gave a sensitivity of 100% and specificity of 79% for subsequent post-traumatic symptoms. In a similar vein, mood profiling was used to investigate effects of stress during basic army combat training (Lieberman et al., 2016). Other applications of mood profiling include monitoring the psychological well-being of cardiac rehabilitation patients (Sties et al., 2014), post-operative adjustment following prostate surgery (Braslis et al., 1995), post-menopausal symptomology (Wyrwich and Yu, 2011), adolescent suicide risk (Gould et al., 2005), and subjective effects of pharmaceuticals (Salzman et al., 1995) and illicit drugs (Weddington et al., 1990). Mood profiling has also played a valuable role in workplace assessment (Morfeld et al., 2007).

Collectively, research points to the utility of mood profiling in both clinical and non-clinical settings. More generally, mood profiles provide a valuable catalyst for discussion between psychologist and client, which may facilitate both early identification of problems and timely resolution (Terry, 1995). An internet-based mood profiling system based on the BRUMS, referred to as the *In The Mood* website (http://www.moodprofiling.com; Lim, 2011; Terry et al., 2013) has been developed. Transcending the barriers of distance and access to expertise, the *In The Mood* website facilitates mood profiling in populations not previously considered.

In summary, several previous studies have demonstrated that mood profiles of athletes often differ from the general population, and explicated how mood responses relate to sports performance (Beedie et al., 2000; Prapavessis, 2000). Three stereotypical mood profiles have previously been identified, termed the iceberg profile (Morgan, 1980), the Everest profile (Terry, 1995), and the inverse iceberg profile (Terry, 1995). Despite several hundred published studies on mood profiling, it remains unknown if distinct mood profile clusters are identifiable beyond the realm of sport and exercise. Hence, the primary objective of the present study was to investigate if relatively consistent mood patterning was evident among the general population using a web-based delivery method to assess mood.

## Methods

### Participants

The total number of participants involved in the study was 6,532 individuals (male = 3,659, female = 2,873) spread over three samples. Sample A included 2,364 participants (male = 1,219, female = 1,145), Sample B included 2,303 participants (male = 1,288, female = 1,015), and Sample C included 1,865 participants (male = 1,152, female = 713). Participants in each sample were aged from 18 to 65 years or older, and reported a range of educational levels and ethnicities. The sociodemographic composition of each sample is detailed in Table 1. The three samples differed significantly by gender, age, education and ethnicity.

**Table 1:** Sociodemographic characteristics of participants.

	**Sample A**		**Sample B**		**Sample C**		
**Source**	***n***	**%**	***n***	**%**	***n***	**%**	**χ^2^**
Total	2,364	100.0	2,303	100.0	1,865	100.0	
Gender							44.07^†^
Male	1,219	51.6	1,288	55.9	1,152	61.8	
Female	1,145	48.4	1,015	44.1	713	38.2	
Age Group (years)							541.47^†^
18–24	1,416	59.9	1,491	64.7	767	41.1	
25–35	356	15.1	420	18.2	277	14.9	
36–45	353	14.9	201	8.7	306	16.4	
46–55	138	5.8	120	5.2	352	18.9	
56–65	87	3.7	54	2.3	163	8.7	
>65	14	0.6	17	0.7	0	0.0	
Education							818.14^†^
< High School	41	1.7	204	8.9	57	3.1	
High School	1,221	51.6	745	32.3	654	35.1	
Trade	0	0.0	0	0.0	96	5.2	
TAFE	0	0.0	0	0.0	107	5.7	
University	709	30.0	896	38.9	571	30.6	
Postgraduate	393	16.6	458	19.8	380	20.4	
Ethnicity							1, 144.61^†^
African	123	5.2	137	5.9	159	8.5	
Asian	136	5.8	0	0.0	0	0.0	
Caucasian	962	40.7	1,628	70.7	1,513	81.1	
Indigenous	39	1.6	19	0.8	12	0.6	
Middle Eastern	81	3.4	68	3.0	23	1.2	
Other	1,023	43.3	451	19.6	158	8.5	

*TAFE, Technical and Further Education (e.g., hospitality, tourism); Trade, qualified tradesperson (e.g., plumber, electrician). TAFE and Trade categories not used in Sample A and Sample B*.

† *p < 0.001*.

### Measures

#### Brunel mood scale (BRUMS)

Mood responses were assessed using the BRUMS (Terry et al., 1999, 2003), a scale of 24 mood descriptors using a standard response timeframe of “How do you feel right now?” Participants rated their mood responses on a 5-point Likert scale of 0 = *not at all*, 1 = *a little*, 2 = *moderately*, 3 = *quite a bit*, and 4 = *extremely*. The BRUMS has six subscales (i.e., anger, confusion, depression, fatigue, tension, and vigor), with four items each. Total subscale scores range from 0 to 16. The BRUMS measurement model was validated using multi-sample confirmatory factor analysis (Terry et al., 2003) across samples of adult students (*n* = 656), adult athletes (*n* = 1,984), young athletes (*n* = 676), and schoolchildren (*n* = 596). Subscales have demonstrated adequate internal consistency, with Cronbach alpha coefficients reported as: tension = 0.74, depression = 0.85, anger = 0.82, vigor = 0.85, fatigue = 0.90, and confusion = 0.83 (Terry et al., 1999). Test-retest reliability coefficients have ranged from 0.26 to 0.53 over a 1-week period (Terry et al., 1999, 2003), which is appropriate for a measure of transient feeling states. The psychometric robustness of the BRUMS makes it an appropriate measure in performance environments and its brevity lends itself well to web-based mood profiling.

#### *In The Mood* website

Development of the *In The Mood* website (Lim, 2011; Terry et al., 2013) was guided by the conceptual framework of Lane and Terry (2000). Once respondents complete the BRUMS, an automated report is generated that interprets scores for the six mood dimensions with reference to normative scores, and a brief summary of the potential influence of obtained mood scores on performance. Raw and standard scores plus a graphical representation of the individual mood profile are presented to respondents, and where appropriate, a series of evidence-based mood regulation strategies corresponding with each mood dimension is provided.

### Procedure

Adult participants (≥ 18 years) were recruited from the general population via the *In The Mood* website (Lim, 2011; Terry et al., 2013). Respondents provided informed consent by clicking on the “I agree” checkbox, which initiated a link to the BRUMS. Alternatively, users could navigate away from the informed consent webpage, or withdraw from the study by clicking “I do not wish to participate, take me away.” Closing the browser window also exited the *In The Mood* website without data collection. Using a snowballing technique, data were collected over a 3-year period, with data downloaded periodically into three separate datasets. The respective data collection periods for Sample A, Sample B, and Sample C were March 2011 to October 2011, November 2011 to October 2013, and November 2013 to May 2014. Lim (2011) showed that mood responses derived from the *In The Mood* website did not differ significantly from data collected using the hardcopy version of the BRUMS. The research was granted ethical approval by the Human Research Ethics Committee at the University of Southern Queensland (H13REA169).

### Data analysis

#### Cluster analysis

Agglomerative, hierarchical cluster analysis was used to distinguish cluster metrics, and k-means clustering with random seeds was used to refine the final parameter solution. Cluster analysis is an exploratory technique designed to delineate natural groups that are undefined *a priori*. Given that hierarchical and partitioning computations will group even random unrelated data (Mooi and Sarstedt, 2011), theoretical considerations are especially salient. Using an iterative procedure, cluster membership is re-evaluated and proximity metrics re-calculated to minimize within-group variance and maximize between-group variance (Everett, 1993). Ward's method was used to determine cluster numbers, followed by the k-means method to fine tune cluster boundaries, as recommended by Clatworthy et al. (2007). All analyses were conducted using the Statistical Package for the Social Sciences, version 23.

#### Data screening

Cases were screened for implausible responses and deleted where identified. Given that all BRUMS questions required a response before data were transferred to the secure database, no missing values were detected. Although significant univariate non-normality was evident for some subscales (e.g., depression), it is typical that the distribution of negative mood scores show large numbers at the lower end of the scale, and smaller numbers at the upper end (Terry et al., 1999). Following visual inspection of the frequency distributions for skewness and kurtosis, it was concluded that deviations from normal distribution were unlikely to make a substantive difference to the analyses, and no trimming of the dataset occurred. A total of 217 multivariate outliers, based on Mahalanobis distance statistics at *p* < 0.001, were identified but scrutiny of individual cases suggested that they were all plausible response patterns. Further, in population studies, scores approaching the extremes of scale ranges are of particular interest when they reflect unusual although legitimate mood responses (Tabachnick and Fidell, 1996). Hence, multivariate outliers were retained in the dataset. The full range of scores from 0 to 16 was evident for each of the BRUMS subscales. Mean T-scores, standard deviations, and 95% confidence intervals for each mood dimension within each sample are provided in Table 2.

**Table 2:** Descriptive statistics for BRUMS subscales.

	**Sample A (*****n*** = **2,364)**			**Sample B (*****n*** = **2,303)**			**Sample C (*****n*** = **1,865)**		
**Subscale**	***M***	***SD***	**95% CI**	***M***	***SD***	**95% CI**	***M***	***SD***	**95% CI**
Tension	46.65	7.81	[46.33, 46.96]	47.24	8.52	[46.89, 47.59]	45.89	8.00	[45.53, 46.25]
Depression	49.95	10.26	[49.54, 50.36]	51.85	11.83	[51.37, 52.33]	51.12	10.82	[50.63, 51.61]
Anger	49.80	8.29	[49.46, 50.13]	52.15	10.28	[51.73, 52.57]	50.89	9.35	[40.47, 51.32]
Vigor	48.59	9.12	[48.22, 48.95]	49.44	9.26	[49.06, 49.82]	49.58	8.92	[49.18, 49.99]
Fatigue	52.26	9.55	[51.88, 52.65]	52.64	9.42	[52.26, 53.03]	52.17	9.38	[51.74, 52.59]
Confusion	49.81	9.53	[49.43, 50.20]	51.72	10.66	[51.29, 52.16]	49.48	9.54	[49.04, 49.91]

*All scores are T-scores*.

## Results

### Identification of mood profile clusters in sample A

Data were analyzed using agglomerative, hierarchical cluster analysis. Ward's method was the chosen clustering algorithm, given theoretical considerations that both the shape and magnitude of the mood profiles would be relevant. Squared euclidean distance was used as the proximity measure to maximize differences between heterogeneous groups. Three checks were conducted to verify the cluster solution (Blashfield, 1980). First, visual examination of the scree plot showed a clear change in trajectory at a 6-cluster solution. Second, review of the final 25 cases of the agglomeration schedule showed a change in distance coefficients at case 2,358. Third, each cluster solution was reviewed, including the member contributions for each possible solution. Five distinct clusters were traced back from step six: H2 (*n* = 109), H3 (*n* = 474), H4 (*n* = 455), H5 (*n* = 302), and H6 (*n* = 630). H1 (*n* = 394) was not as stable as the other five clusters, in that H1 (*n* = 284) and H7 (*n* = 110) combined immediately before the sixth step. However, given that the scree plot showed a distinct elbow, and distance coefficients increased from case 2,358, a 6-cluster solution was judged to provide the best fit to the data.

Inter-correlations among the six clusters were examined to evaluate the extent to which clusters were mutually exclusive. Given the large sample size, even small correlations reached statistical significance and hence a criterion of < 0.70 was applied to signify that inter-correlations represented less than half of the covariance and were therefore indicative of substantial independence. Large negative correlations between H2 and H4, H5, and H6 represented reverse cluster patterning rather than denoting similarity. A strong positive relationship was found between cluster H1 and H3, with 81.0% shared variance. Additionally, H4, H5, and H6 were also found to be closely related, sharing 84.6–92.2% common variance. These inter-relationships suggested homogeneous clusters. Despite some clusters sharing a similar shape, mean scores for the six mood dimensions were sufficiently different to satisfy the criterion of heterogeneous groups according to a Ward's analysis.

Following the initial identification of the six clusters, a partitioning method was used to validate the findings, and further refine the final parameter solution. K-means clustering was conducted using random aggregation centers with a prescribed 6-cluster solution. The hierarchical and k-means techniques both produced clusters that pooled a large proportion of shared variance (see Table 3). Additionally, the inter-correlations between the prescribed k-means solution yielded a similar result to the inter-correlations from the hierarchical cluster analysis. Large negative correlations were found between K2 and K3, K4, and K6. A positive relationship was found between K1 and K5 with 82.8% common variance, while clusters K3, K4, and K6 were also related to one another sharing 88.4% to 90.3% common variance. Taken together, these findings provided strong evidence that the cluster structures were both independent and stable.

**Table 3:** Inter-correlation matrix of the hierarchical and K-means clusters (*n* = 2,364).

	**Hierarchical**					
**K-means**	**H1**	**H2**	**H3**	**H4**	**H5**	**H6**
K1	0.97	0.18	0.89	0.27	0.38	0.15
K2	−0.03	1.00	−0.06	−0.89	−0.80	−0.93
K3	0.57	−0.83	0.50	0.99	0.93	0.94
K4	0.37	−0.94	0.41	0.97	0.96	1.00
K5	0.90	0.02	0.99	0.33	0.55	0.31
K6	0.67	−0.76	0.70	0.93	0.99	0.93

*H1, H2, … H6 denotes hierarchical clusters identified in Sample A. K1, K2, … K6 denotes k-means clusters identified in Sample A. H1 (n = 394), H2 (n = 109), H3 (n = 474), H4 (n = 455), H5 (n = 302), H6 (n = 630). K1 (n = 244), K2 (n = 64), K3 (n = 349), K4 (n = 695), K5 (n = 409), K6 (n = 603)*.

Cluster 1 was previously identified in the literature as the *inverse iceberg* profile (Terry, 1995), characterized by low vigor, together with high tension, depression, anger, fatigue, and confusion. Cluster 2, a novel mood profile, was termed the *inverse Everest* profile, characterized by low vigor, together with high tension and fatigue, and very high depression, anger, and confusion. Cluster 3, a second novel mood profile, was termed the *surface* profile, characterized by slightly above average levels of tension, depression, anger, vigor, fatigue, and confusion. Cluster 4 was the classic *iceberg* profile (Morgan, 1980), characterized by high vigor, together with low tension, depression, anger, fatigue, and confusion. Cluster 5, a third novel mood profile, was termed the *shark fin* profile, characterized by low tension, depression, anger, vigor, and confusion together with high fatigue. Finally, cluster 6, a fourth novel mood profile, was termed the *submerged* profile, characterized by low scores for tension, depression, anger, vigor, fatigue, and confusion. Table 4 includes descriptive statistics for the 6-cluster solution in Sample A.

**Table 4:** Descriptive statistics of the 6-cluster solution in Sample A (*n* = 2,364).

	**Iceberg profile (*****n*** = **695)**			**Inverse Everest profile (*****n*** = **64)**			**Inverse iceberg profile (*****n*** = **244)**		
**Mood dimension**	***M***	***SD***	**95% CI**	***M***	***SD***	**95% CI**	***M***	***SD***	**95% CI**
Tension	42.84	3.59	[42.58, 43.11]	67.70	8.64	[65.54, 69.86]	56.65	7.64	[55.69, 57.61]
Depression	44.98	2.58	[44.79, 45.17]	87.17	11.95	[84.19, 90.16]	63.86	9.95	[62.61, 65.11]
Anger	46.26	2.69	[46.06, 46.47]	79.05	10.81	[76.35, 81.75]	59.82	9.20	[58.66, 60.98]
Vigor	57.33	5.32	[56.93, 57.73]	42.50	10.64	[39.84, 45.16]	45.73	7.54	[44.77, 46.68]
Fatigue	45.72	4.69	[45.37, 46.07]	68.80	7.23	[67.02, 70.58]	60.80	8.38	[59.74, 61.85]
Confusion	44.80	3.38	[44.55, 45.05]	80.39	11.22	[77.59, 83.19]	63.20	8.23	[62.16, 64.24]
	**Shark fin profile (*****n*** = **409)**			**Submerged profile (*****n*** = **603)**			**Surface profile (*****n*** = **349)**		
Tension	44.42	5.23	[43.91, 44.92]	43.23	4.18	[42.89, 43.56]	51.90	6.10	[51.26, 52.54]
Depression	48.97	6.67	[48.32, 49.62]	46.34	4.75	[45.96, 46.72]	50.68	7.14	[49.93, 51.43]
Anger	48.00	4.58	[47.55, 48.45]	46.50	3.14	[46.25, 46.75]	52.26	7.02	[51.52, 53.00]
Vigor	41.12	6.58	[40.48, 41.76]	42.52	4.67	[42.15, 42.89]	53.51	6.34	[52.85, 54.18]
Fatigue	64.16	6.22	[63.55, 64.76]	46.99	4.51	[46.63, 47.35]	51.46	5.85	[50.84, 52.07]
Confusion	47.47	5.59	[46.93, 48.02]	45.99	4.65	[45.61, 46.36]	54.20	7.16	[53.44, 54.95]

A *post-hoc* discriminant function analysis (DFA) was used to calculate the extent to which cases could be correctly classified into clusters. DFA is a two-step statistical procedure that involves significance testing of discriminant functions followed by calculation of correctly classified cases. The ratio of cases to independent variables was 394 to 1, which far exceeded the requirement of ≥ 20 to 1. The number of cases in the smallest cluster was 64, which exceeded the preferred number of cases (i.e., ≥ 20) per group. Five discriminant functions collectively accounted for 100% of the variance, and each function predicted significant variance (see Table 5).

**Table 5:** Discriminant functions for Sample A (*n* = 2,364), Sample B (*n* = 2,303), and Sample C (*n* = 1,865).

**Function**	**Eigenvalue**	**% of Variance**	**Cumulative %**	**Canonical correlation**
**SAMPLE A**				
1	5.678	71.3	71.3	0.922
2	1.693	21.3	92.5	0.793
3	0.498	6.2	98.8	0.576
4	0.094	1.2	99.9	0.293
5	0.004	0.1	100.0	0.067
**SAMPLE B**				
1	6.607	76.3	76.3	0.932
2	1.558	18.0	94.3	0.780
3	0.393	4.5	98.8	0.531
4	0.099	1.1	99.9	0.300
5	0.004	0.1	100.0	0.065
**SAMPLE C**				
1	6.739	77.4	77.4	0.933
2	1.475	16.9	94.3	0.772
3	0.438	5.0	99.4	0.552
4	0.051	0.6	99.9	0.220
5	0.005	0.1	100.0	0.073

In line with the cut-off criterion, only predictor variables with loadings of ± 0.30 were interpreted. Based on the structure matrix (see Table 6), mood dimensions that associated with DF^1A^ included high levels of confusion, fatigue, tension, depression, and anger. Variables associated with DF^2A^ included high levels of vigor, and low levels of fatigue, whereas those associated with DF^3A^ included high levels of vigor *and* fatigue. Variables associated with DF^4A^ included low levels of tension and high levels of depression, and those associated with DF^5A^ included low levels of confusion and depression, and a high level of anger.

**Table 6:** Structure matrix and unstandardized canonical coefficients for Sample A (*n* = 2,364), Sample B (*n* = 2,303), and Sample C (*n* = 1,865).

	**Structure matrix**														
**Mood dimension**	**DF^1A^**	**DF^2A^**	**DF^3A^**	**DF^4A^**	**DF^5A^**	**DF^1B^**	**DF^2B^**	**DF^3B^**	**DF^4B^**	**DF^5B^**	**DF^1C^**	**DF^2C^**	**DF^3C^**	**DF^4C^**	**DF^5C^**
Tension	0.445	0.268	−0.063	−0.691^*^	−0.126	0.500	0.270	0.032	−0.647^*^	0.485	0.478	0.257	−0.075	0.604^*^	0.432
Depression	0.560	0.149	−0.169	0.673^*^	−0.303	0.630^*^	0.169	−0.354	0.593	0.298	0.570	0.145	−0.130	−0.727^*^	0.119
Anger	0.494	0.234	−0.114	0.259	0.781^*^	0.487	0.225	−0.112	0.220	−0.521^*^	0.520^*^	0.254	−0.228	−0.225	0.247
Vigor	−0.176	0.728^*^	0.663	0.012	0.015	−0.139	0.700^*^	0.671	0.187	−0.052	−0.141	0.756^*^	0.612	−0.025	−0.032
Fatigue	0.444	−0.545	0.706^*^	−0.082	0.015	0.449	−0.590	0.659^*^	0.110	0.052	0.438	−0.531	0.715^*^	0.029	0.001
Confusion	0.546^*^	0.250	−0.155	−0.255	−0.404	0.560^*^	0.211	−0.039	−0.255	−0.412	0.564	0.220	−0.152	0.281	−0.712^*^
	**Unstandardised canonical coefficients**														
Tension	0.178	0.108	−0.059	−0.341	0.007	0.194	0.128	0.012	−0.353	0.285	0.187	0.115	−0.008	0.316	0.355
Depression	0.255	0.089	−0.026	0.451	−0.304	0.252	0.121	−0.164	0.467	0.352	0.222	0.100	0.023	−0.520	0.082
Anger	0.254	0.118	−0.078	0.044	0.571	0.178	0.044	−0.069	−0.011	−0.341	0.237	0.070	−0.187	0.019	0.134
Vigor	−0.064	0.301	0.278	0.049	−0.026	−0.047	0.274	0.258	0.107	0.030	−0.055	0.312	0.265	−0.036	−0.020
Fatigue	0.182	−0.239	0.311	−0.013	0.005	0.180	−0.273	0.306	0.060	0.023	0.189	−0.236	0.321	0.022	0.005
Confusion	0.234	0.122	−0.075	−0.112	−0.228	0.210	0.089	0.003	−0.145	−0.329	0.267	0.105	−0.090	0.153	−0.594

* *Largest absolute correlation between each variable and any discriminant function*.

*DF^1^, DF^2^, … DF^5^ denotes discriminant functions. ^ABC^ denotes Sample A, Sample B, and Sample C, respectively*.

DFA showed cluster membership to be classified correctly with a high degree of accuracy for all clusters (see Table 7). Prior probabilities were 10.3, 2.7, 14.8, 29.4, 17.3, and 25.5% for the inverse iceberg profile, inverse Everest profile, surface profile, iceberg profile, shark fin profile, and submerged profile, respectively. The proportional by chance accuracy rate was computed by squaring and summing the proportion of cases in each group from the table of prior probabilities for groups (i.e., 0.103^2^ + 0.027^2^ + 0.148^2^ + 0.294^2^ + 0.173^2^ + 0.255^2^ = 0.215). Additionally, when the discriminant functions were used to predict group membership, the hit ratio was very high. A total of 95.2% of cases were correctly reclassified back into the original categories. This percentage was notably higher than the minimum classification accuracy rate of 46.5% (i.e., the proportional by chance accuracy rate + 25%), suggesting that the overlap of the distributions was small, and the function was a good discriminator between groups.

**Table 7:** Classifications for Sample A (*n* = 2,364), Sample B (*n* = 2,303), and Sample C (*n* = 1,865).

	**Predicted group membership**							
**Cluster**	**1**	**2**	**3**	**4**	**5**	**6**	***N***	**%**
**SAMPLE A**								
Iceberg	695	0	0	0	0	0	695	100.0
Inverse Everest	0	63	1	0	0	0	64	98.4
Inverse Iceberg	0	0	225	7	0	12	244	92.2
Shark Fin	4	0	1	386	17	1	409	94.4
Submerged	0	0	2	0	593	8	603	98.3
Surface	35	0	10	5	10	289	349	82.8
**SAMPLE B**								
Iceberg	684	0	0	1	1	0	686	99.7
Inverse Everest	0	76	7	0	0	0	83	91.6
Inverse Iceberg	0	1	273	1	0	9	284	96.1
Shark Fin	0	0	1	289	26	2	318	90.9
Submerged	15	0	0	0	565	6	586	96.4
Surface	34	0	4	2	12	294	346	85.0
**SAMPLE C**								
Iceberg	519	0	0	0	3	1	523	99.2
Inverse Everest	0	41	3	0	0	0	44	93.2
Inverse Iceberg	0	0	171	1	0	2	174	98.3
Shark Fin	1	0	0	286	18	2	307	93.2
Submerged	10	0	0	0	531	0	541	98.2
Surface	18	0	4	9	17	228	276	82.6

*1, Iceberg Profile; 2, Inverse Everest Profile; 3, Inverse Iceberg Profile; 4, Shark Fin Profile; 5, Submerged Profile; 6, Surface Profile*.

### Replication of mood profile clusters—sample B and sample C

K-means clustering using random aggregation centers and a prescribed 6-cluster solution was used to replicate the findings derived from Sample A. Mean T-scores of the cluster centroids for each mood dimension in each sample are presented in Table 8.

**Table 8:** Cluster centroids for Sample A (*n* = 2,364), Sample B (*n* = 2,303), and Sample C (*n* = 1,865).

	**Cluster**					
**Mood dimension**	**1**	**2**	**3**	**4**	**5**	**6**
**SAMPLE A**						
Tension	42.84	67.70	56.65	44.42	43.23	51.90
Depression	44.98	87.17	63.86	48.97	46.34	50.68
Anger	46.26	79.05	59.82	48.00	46.50	52.26
Vigor	57.33	42.50	45.73	41.12	42.52	53.51
Fatigue	45.72	68.80	60.80	64.16	46.99	51.46
Confusion	44.80	80.39	63.20	47.47	45.99	54.20
**SAMPLE B**						
Tension	42.33	66.76	59.16	45.22	42.91	51.72
Depression	45.22	89.53	67.97	50.09	47.26	52.10
Anger	47.24	81.71	64.87	50.15	47.26	54.49
Vigor	57.13	42.71	47.25	42.59	42.51	55.66
Fatigue	45.11	67.59	61.45	65.11	49.21	51.11
Confusion	45.27	80.67	66.09	50.39	46.55	55.77
**SAMPLE C**						
Tension	42.25	70.45	57.82	44.97	41.67	50.64
Depression	45.65	87.43	69.87	52.00	45.95	53.03
Anger	46.64	81.86	66.69	49.48	46.66	53.92
Vigor	58.84	45.39	46.90	42.35	44.58	52.26
Fatigue	45.69	70.00	60.91	64.10	47.02	52.92
Confusion	44.42	78.73	66.05	48.98	44.62	54.00

*1, Iceberg Profile; 2, Inverse Everest Profile; 3, Inverse Iceberg Profile; 4, Shark Fin Profile; 5, Submerged Profile; 6, Surface Profile*.

The same six mood profile clusters identified in Sample A (i.e., iceberg, inverse Everest, inverse iceberg, shark fin, submerged, and surface profiles) were also evident in the other two samples. Descriptive statistics and cluster sizes for Sample B and Sample C are shown in Tables 9, 10, respectively. The smallest cluster had 83 cases in Sample B and 44 cases in Sample C, exceeding the minimum threshold of 20. The five discriminant functions extracted accounted for 100% of the variance in both samples, and each function predicted significant variance (see Table 5).

**Table 9:** Descriptive statistics of the 6-cluster solution in Sample B (*n* = 2,303).

	**Iceberg profile (*****n*** = **686)**			**Inverse Everest profile (*****n*** = **83)**			**Inverse iceberg profile (*****n*** = **284)**		
**Mood dimension**	***M***	***SD***	**95% CI**	***M***	***SD***	**95% CI**	***M***	***SD***	**95% CI**
Tension	42.33	3.28	[42.08, 42.57]	66.76	9.75	[64.63, 68.89]	59.16	7.01	[58.34, 59.98]
Depression	45.22	2.85	[45.01, 45.44]	89.53	10.30	[87.28, 91.78]	67.97	9.57	[66.85, 69.09]
Anger	47.24	4.68	[46.89, 47.59]	81.71	11.18	[79.27, 84.15]	64.87	8.83	[63.84, 65.90]
Vigor	57.13	5.41	[56.72, 57.53]	42.71	10.52	[40.41, 45.01]	47.25	8.01	[46.31, 48.18]
Fatigue	45.11	4.66	[44.76, 45.46]	67.59	7.54	[65.94, 69.24]	61.45	7.15	[60.62, 62.29]
Confusion	45.27	3.61	[45.00, 45.54]	80.67	10.00	[78.49, 82.86]	66.09	7.97	[65.16, 67.02]
	**Shark fin profile (*****n*** = **318)**			**Submerged profile (*****n*** = **586)**			**Surface profile (*****n*** = **346)**		
Tension	45.22	5.06	[44.67, 45.78]	42.91	3.71	[42.61, 43.21]	51.72	6.44	[51.04, 52.40]
Depression	50.09	6.57	[49.37, 50.82]	47.26	5.41	[46.82, 47.70]	52.10	6.84	[51.37, 52.82]
Anger	50.15	6.77	[49.40, 50.90]	47.26	4.02	[46.93, 47.58]	54.49	7.59	[53.68, 55.29]
Vigor	42.59	7.44	[41.77, 43.41]	42.51	5.17	[42.09, 42.93]	55.66	6.59	[54.97, 56.36]
Fatigue	65.11	5.88	[64.46, 65.76]	49.21	4.79	[48.82, 49.60]	51.11	5.12	[50.57, 51.65]
Confusion	50.39	6.92	[49.62, 51.15]	46.55	4.89	[46.16, 46.95]	55.77	7.37	[54.99, 56.55]

**Table 10:** Descriptive statistics of the 6-cluster solution in Sample C (*n* = 1,865).

	**Iceberg profile (*****n*** = **523)**			**Inverse Everest profile (*****n*** = **44)**			**Inverse iceberg profile (*****n*** = **174)**		
**Mood dimension**	***M***	***SD***	**95% CI**	***M***	***SD***	**95% CI**	***M***	***SD***	**95% CI**
Tension	42.25	3.20	[41.97, 42.52]	70.45	7.32	[68.23, 72.68]	57.82	6.81	[56.80, 58.84]
Depression	45.65	3.69	[45.33, 45.97]	87.43	11.91	[83.81, 91.05]	69.87	9.03	[68.52, 71.22]
Anger	46.64	3.27	[46.36, 46.92]	81.86	9.86	[78.87, 84.86]	66.69	9.40	[65.28, 68.10]
Vigor	58.84	4.94	[58.42, 59.27]	45.39	8.67	[42.75, 48.02]	46.90	7.95	[45.71, 48.09]
Fatigue	45.69	4.72	[45.28, 46.09]	70.00	6.59	[68.00, 72.00]	60.91	7.21	[59.83, 61.99]
Confusion	44.42	2.73	[44.19, 44.66]	78.73	9.26	[75.91, 81.54]	66.05	8.61	[64.76, 67.34]
	**Shark fin profile (*****n*** = **307)**			**Submerged profile (*****n*** = **541)**			**Surface profile (*****n*** = **276)**		
Tension	44.97	5.67	[44.33, 45.61]	41.67	2.84	[41.43, 41.91]	50.64	7.31	[49.77, 51.51]
Depression	52.00	7.68	[51.14, 52.87]	45.95	3.95	[45.61, 46.28]	53.03	7.13	[52.18, 53.87]
Anger	49.48	5.33	[48.88, 50.07]	46.66	3.19	[46.39, 46.93]	53.92	7.57	[53.03, 54.82]
Vigor	42.35	6.37	[41.63, 43.06]	44.58	5.96	[44.07, 45.08]	52.26	6.38	[51.50, 53.02]
Fatigue	64.10	6.43	[63.38, 64.82]	47.02	4.68	[46.62, 47.41]	52.92	5.83	[52.23, 53.61]
Confusion	48.98	6.35	[48.26, 49.69]	44.62	3.25	[44.35, 44.90]	54.00	7.16	[53.15, 54.84]

Based on the structure matrix for Sample B, the mood dimensions strongly associated with DF^1B^ included high levels of depression, confusion, tension, anger, and fatigue. The predictor variables strongly associated with DF^2B^ included a high level of vigor and low fatigue. The predictor variables strongly associated with DF^3B^ included a high level of vigor and fatigue, together with a low level of depression. The predictor variables strongly associated with DF^4B^ included low tension and high depression, while the predictor variables strongly associated with DF^5B^ included low levels of anger and confusion, and a high level of tension. Based on the structure matrix for Sample C, the mood dimensions strongly associated with DF^1C^ included high levels of anger, fatigue, depression, tension, and confusion. The predictor variables strongly associated with DF^2C^ included a high level of vigor and low fatigue. The predictor variables strongly associated with DF^3C^ included high levels of fatigue and vigor. The predictor variables strongly associated with DF^4C^ included low levels of depression and high tension, while the predictor variables strongly associated with DF^5C^ included high tension and low levels of confusion. The structure matrix and unstandardized canonical coefficients for each sample are shown in Table 6.

The DFA showed that cluster membership was correctly classified with a high degree of accuracy for both Sample B and Sample C. Prior probabilities for Sample B and Sample C respectively were 12.3, 3.6, 15.0, 29.8, 13.8, 25.4% and 9.3, 2.4, 14.8, 28.0, 16.5, 29.0%, for the inverse iceberg profile, inverse Everest profile, surface profile, iceberg profile, shark fin profile, and submerged profile. The proportional by chance accuracy rates were also computed (i.e., 0.123^2^ + 0.036^2^ + 0.150^2^ + 0.298^2^ + 0.138^2^ + 0.254^2^ = 0.211 and 0.093^2^ + 0.024^2^ + 0.148^2^ + 0.280^2^ + 0.165^2^ + 0.290^2^ = 0.221, respectively).

**Figure 1:**
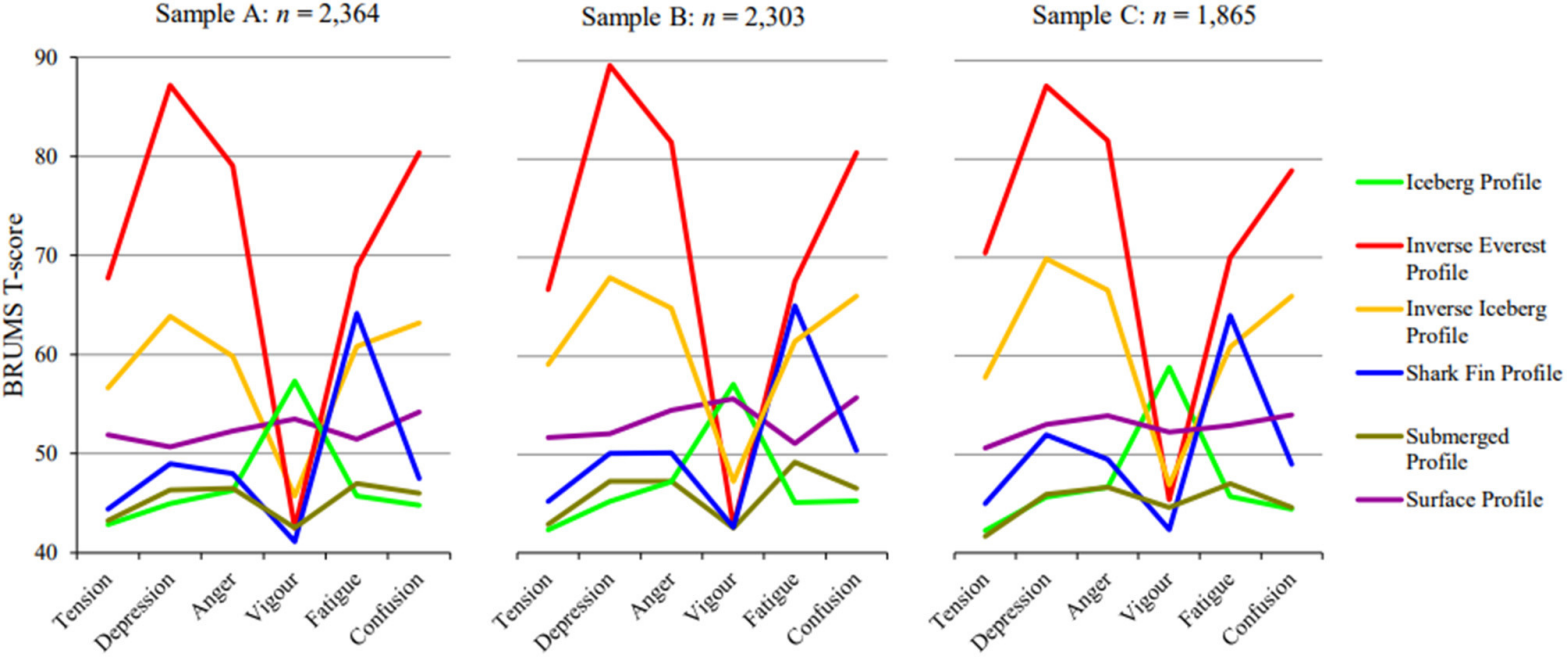
Visual summary of the 6-cluster solutions in three samples.

Additionally, when the discriminant functions were used to predict group membership, the hit ratio was very high for both samples. A total of 94.7 and 95.2% of the cases were correctly reclassified back into the original categories for Sample B and Sample C, respectively. These percentages were notably higher than the minimum classification accuracy rate of 46.1% for Sample B, and 47.1% for Sample C. These findings indicate that overlap of distributions was small, and functions within each sample were good discriminators between groups. Table 11 lists the classification function coefficients for each sample. Overall, the k-means cluster analyses for Sample B and Sample C produced cluster structures that were very similar to those identified in Sample A. A visual summary of the 6-cluster solution across samples is provided in Figure 1.

**Table 11:** Classification function coefficients for Sample A (*n* = 2,364), Sample B (*n* = 2,303), and Sample C (*n* = 1,865).

	**Cluster**					
**Mood dimension**	**1**	**2**	**3**	**4**	**5**	**6**
**SAMPLE A**						
Tension	0.102	2.023	1.201	0.227	0.198	0.878
Depression	0.378	3.575	1.734	0.584	0.328	0.651
Anger	0.006	3.186	1.351	0.206	0.085	0.698
Vigor	1.852	0.945	1.094	0.753	0.829	1.571
Fatigue	0.458	2.116	1.551	1.859	0.540	0.854
Confusion	0.158	2.838	1.550	0.306	0.237	0.875
**SAMPLE B**						
Tension	0.161	1.938	1.484	0.324	0.203	0.964
Depression	0.441	3.442	1.830	0.509	0.393	0.677
Anger	−0.212	1.793	0.859	0.091	−0.064	0.290
Vigor	1.642	0.854	1.079	0.769	0.750	1.539
Fatigue	0.447	2.083	1.673	2.086	0.773	0.885
Confusion	0.169	2.358	1.461	0.488	0.235	0.890
**SAMPLE C**						
Tension	0.285	2.548	1.468	0.472	0.189	0.994
Depression	0.506	3.223	1.997	0.769	0.314	0.771
Anger	−0.283	2.715	1.480	0.095	−0.065	0.476
Vigor	1.942	1.060	1.145	0.835	0.953	1.484
Fatigue	0.473	2.373	1.668	1.957	0.577	1.055
Confusion	0.091	3.071	2.076	0.496	0.133	0.970

*1, Iceberg Profile; 2, Inverse Everest Profile; 3, Inverse Iceberg Profile; 4, Shark Fin Profile; 5, Submerged Profile; 6, Surface Profile*.

Cluster scores were consistent across the three samples, with mean values for the various profiles constrained within relatively narrow bounds (see Table 8). Additionally, the percentage of participants in each cluster was very similar across all three samples: inverse iceberg ~10.6% (range = 9.3–12.3%), inverse Everest ~2.9% (range = 2.4–3.6%), surface ~14.9% (range = 14.8–15.0%), iceberg ~29.1% (range = 28.0–29.8%), shark fin ~15.9% (range = 13.8–17.3%), and submerged ~26.6% (range = 25.4–29.0%) profile. Finally, the percentage of correct classification of cluster membership also showed little variation: inverse iceberg (range = 92.2–98.3%), inverse Everest (range = 91.6–98.4%), surface (range = 82.6–85.0%), iceberg (range = 99.2–100%), shark fin (range = 90.9–94.4%), and submerged (range = 96.4–98.3%) profile.

### Sociodemographic distribution of mood profiles

Chi-squared tests of goodness-of-fit were used to assess the distribution of mood profiles according to the gender, age and level of education of participants. The distribution of inverse Everest and surface profiles was independent of gender in all three samples (see Table 12). Females were significantly over-represented for the inverse iceberg profile in all three samples and for the shark fin profile in two samples. Conversely, males were significantly over-represented for the iceberg profile in all three samples. No clear trend was evident for the submerged profile.

**Table 12:** Distribution of mood profile clusters by gender, age, education, and ethnicity.

	**Cluster**					
**Source**	**1**	**2**	**3**	**4**	**5**	**6**
**GENDER**						
Male^A^	406^†^^+^	33	107^*^^−^	196	288^*^^−^	189
Female^A^	289^†^^−^	31	137^*^^+^	213	315^*^^+^	160
Male^B^	431^†^^+^	51	139^*^^−^	150^†^^−^	318	199
Female^B^	255^†^^−^	32	145^*^^+^	168^†^^+^	268	147
Male^C^	381^†^^+^	24	92^*^^−^	137^†^^−^	357^*^^+^	161
Female^C^	142^†^^−^	20	82^*^^+^	170^†^^+^	184^*^^−^	115
**AGE GROUP (YR.)**						
18–24^A^	358^†^^−^	29^*^^−^	151	274^§^^+^	374	230^*^^+^
25–35^A^	110	22^†^^+^	33	54	89	48
36–45^A^	138^†^^+^	7	35	55	79	39^*^^−^
46–55^A^	46	3	19	15^*^^−^	35	20
56–65^A^	38^§^^+^	1	5	10	24	9
> 65^A^	5	2^§^^+^	1	1	2	3
18–24^B^	448	38^†^^−^	173	226^*^^+^	374	232
25–35^B^	94^†^^−^	24^§^^+^	68^§^^+^	59	112	63
36–45^B^	67	10	15^*^^−^	22	61	26
46–55^B^	45	6	17	10	27	15
56–65^B^	23^*^^+^	2	10	1^§^^−^	8	10
> 65^B^	9^*^^+^	3^§^^+^	1	0	4	0
18–24^C^	223	15	55^§^^−^	131	225	118
25–35^C^	52^†^^−^	9	40^§^^+^	63^§^^+^	72	41
36–45^C^	87	8	26	44	99	42
46–55^C^	112	8	31	50	104	47
56–65^C^	49	4	22	19	41	28
**EDUCATION**						
<High School^A^	4^§^^−^	1	8^*^^+^	5	20^†^^+^	3
High School^A^	337^*^^−^	25^*^^−^	124	235^§^^+^	317	183
University^A^	233^*^^+^	22	64	116	178	96
Postgraduate^A^	121	16	48	53^*^^−^	88	67
<High School^B^	52	8	32	23	55	34
High School^B^	255^§^^+^	21	76^*^^−^	101	184	108
University^B^	243^*^^−^	31	102	149^§^^+^	234	137
Postgraduate^B^	136	23	74^§^^+^	45^§^^−^	113	67
<High School^C^	19	0	5	10	18	5
High School^C^	202^*^^+^	15	42^§^^−^	110	187	98
TAFE^C^	20^*^^−^	6^*^^+^	23^†^^+^	17	26	15
Trade^C^	28	1	15^*^^+^	16	26	10
University^C^	165	12	49	89	170	86
Postgraduate^C^	89^*^^−^	10	40	65	114	62
**ETHNICITY**						
African^A^	56^†^^+^	0	7	19	32	9^*^^−^
Asian^A^	33	9^§^^+^	22^*^^+^	9^†^^−^	43	20
Caucasian^A^	263	32	117^*^^+^	169	216^§^^−^	165^§^^+^
Indigenous^A^	20^§^^+^	0	2	6	9	2
Middle Eastern^A^	41^†^^+^	2	8	11	14	5^*^^−^
Other^A^	282	21	88^*^^−^	195^*^^+^	289^§^^+^	148
African^B^	37	7	14	26	35	18
Caucasian^B^	515^§^^+^	52	179^§^^−^	214	443^§^^+^	225^*^^−^
Indigenous^B^	1^*^^−^	3^§^^+^	6^§^^+^	1	3	5
Middle Eastern^B^	21	7^§^^+^	12	4	7^§^^−^	17^*^^+^
Other^B^	112^§^^−^	14	73^§^^+^	73	98^*^^−^	81
African^C^	60^§^^+^	3	12	14^§^^−^	59^*^^+^	11^§^^−^
Caucasian^C^	403^§^^−^	38	140	273^†^^+^	427	232
Indigenous^C^	4	0	4^§^^+^	1	2	1
Middle Eastern^C^	12^§^^+^	2^*^^+^	1	0^*^^−^	6	2
Other^C^	44	1	17	19	47	30

*1, Iceberg Profile; 2, Inverse Everest Profile; 3, Inverse Iceberg Profile; 4, Shark Fin Profile; 5, Submerged Profile; 6, Surface Profile. TAFE, Technical and Further Education (e.g., hospitality, tourism); Trade, qualified tradesperson (e.g., plumber, electrician)*.

A *Sample A (n = 2,364)*.

B *Sample B (n = 2,303)*.

C *Sample C (n = 1,865)*.

+ , over-represented;

− *, under-represented*.

* *p < 0.05*,

§ *p < 0.01*,

† *p < 0.001*.

For age groupings, distribution of the submerged profile was independent of age in all three samples. Similarly, in Sample B and Sample C, the surface profile was independent of age grouping. Participants aged 18–24 were significantly over-represented for the shark fin profile and significantly under-represented for the inverse Everest profile in both Sample A and Sample B. Those aged 25–35 were significantly over-represented for the inverse Everest profile in Sample A and Sample B, significantly over-represented for the inverse iceberg in Sample B and Sample C, and significantly under-represented for the iceberg profile in Sample B and Sample C. Those aged 56–65 were significantly over-represented for the iceberg profile in Sample A and Sample B.

For level of education, distribution of the surface profile was independent in all three samples. Similarly, the submerged profile was independent of education level in Sample B and Sample C. High school certificate participants were significantly over-represented for the iceberg profile and significantly under-represented for the inverse iceberg profile in both Sample B and Sample C. Postgraduate participants were significantly under-represented for the shark fin profile in Sample A and Sample B. Those with a TAFE certificate were significantly over-represented for the inverse Everest and inverse iceberg profiles and significantly under-represented for the iceberg profile in Sample C. Finally, those with a trade qualification were significantly over-represented for the inverse iceberg profile in Sample C.

For ethnicity, African participants were significantly over-represented for the iceberg profile and significantly under-represented for the surface profile in both Sample A and Sample C. Asian participants were significantly over-represented for the inverse Everest and inverse iceberg profiles and significantly under-represented for the shark fin profile in Sample A. Indigenous participants were significantly over-represented for the iceberg profile in Sample A and significantly over-represented for the inverse iceberg profile in Sample B and Sample C. Middle Eastern participants were significantly over-represented for the iceberg profile in Sample A and Sample C. Caucasian participants, who formed the largest proportion of the total sample, showed several significant deviations from the expected distribution across mood profiles (Table 12) but no clear tends were evident. The sociodemographic trends reported above should be treated with caution due to violations of the underlying assumption of minimum cell counts for some categories of participants.

## Discussion

Three distinct mood profiles (iceberg, inverse iceberg, and Everest profiles) were previously identified within athletic samples (Morgan, 1980; Terry, 1995). We investigated whether relatively consistent mood patterns were evident within the general population using a web-based delivery method. Three datasets gathered via the *In The Mood* website were interrogated using cluster analytic methodology. More specifically, the mood responses of Sample A (*n* = 2,364) were analyzed using a two-step clustering procedure. Six mood profiles were identified, including two established profiles (i.e., iceberg, inverse iceberg profiles) and four novel profiles (i.e., inverse Everest, shark fin, submerged, and surface profiles). Cluster membership was correctly classified with a high degree of accuracy. Results were replicated in Sample B (*n* = 2,303) and Sample C (*n* = 1,865). Chi-squared tests of goodness-of-fit indicated that the distributions of sociodemographic variables (i.e., gender, age, education level, and ethnicity) across the six mood profiles were significantly different from expected cell counts in all three samples.

These findings raise many research questions worthy of future investigation relating to the antecedents, correlates and consequences of the four novel mood profiles, as well as the profiles previously identified in the literature. The iceberg and Everest profiles have long been associated with healthy cognitive functioning and high to superior levels of physical performance (Morgan, 1980, 1985; Terry, 1995). Despite the longstanding line of investigation into the effects of mood in sporting and to a lesser extent educational contexts, far less is known about how mood affects human functioning in other domains. Hence, there is much scope for assessing the influence of mood profiles generally, and the potentially beneficial effects of the iceberg and Everest profiles in particular, in other intense performance environments, such as in medical, military, business, construction, and mining contexts.

The inverse iceberg profile has frequently been associated with debilitating conditions among athletes, including overtraining syndrome (Budgett, 1998), risk of eating disorders (Terry and Galambos, 2004) and reduced physical performance (Lahart et al., 2013). Our finding that ~11% of the general population reported an inverse iceberg profile suggests that its prevalence is sufficient to warrant further investigations of associated risks and consequences in a range of environments beyond sport, exemplified by van Wijk's et al. (2013) use of mood profiling to screen for risk of post-traumatic stress in military populations. By extension, the inverse Everest profile, a novel mood profile reported by about 3% of our combined sample and representing the most negative of the six mood profiles, may be indicative of clinical psychopathology. High scores for tension and fatigue, combined with very high scores for depression, anger and confusion, represents a profile that shares many of the symptoms of clinically diagnosable mental health conditions. Mood disorder severity occurs along a continuum, with increased symptomology corresponding with greater cognitive deficits, such as distorted thinking, reduced concentration, distractibility, slower reaction time, memory loss, and indecision (Sarapas et al., 2012). The inverse Everest profile would therefore likely associate with a broader range of negative cognitive and behavioral outcomes than previously demonstrated for the inverse iceberg profile, including debilitated performance. Confirming such associations empirically is a clear line of enquiry for future investigations.

The influence on human functioning of the shark fin profile, the second of the novel mood profiles, is also unknown. Notably, the shark fin profile lacks some markers of negative mood, such as high levels of tension, depression, anger, and confusion. It is reasonable to speculate, however, that a profile with the lowest vigor scores of all the profiles, combined with higher fatigue scores than any profile except the inverse Everest (see Figure 1) would have deleterious effects on functioning, particularly in environments requiring energy and alertness. The combination of high fatigue and low vigor is a well-established concern for patient safety in clinical health environments (Gaba and Howard, 2002), for road safety (Summala and Mikkola, 1994), and the safety of pilots and passengers in the aviation industry (Bourgeois-Bougrine et al., 2003; Jackson and Earl, 2006). Therefore, future investigations are needed to assess whether the shark fin profile is a potential contributor to accidents caused by impaired functioning in high-risk environments.

A third novel mood profile, the submerged profile, shares many characteristics of the iceberg profile, with below average scores for tension, depression, anger, fatigue, and confusion. The sole difference between the submerged and iceberg profiles is the stark contrast in vigor scores, which sit about 15 percentile points (1.5 standard deviations) apart. Hence, the submerged profile is characterized by being relatively devoid of emotion, including vigor, which may well be described as feeling *flat*. Such a mood profile may impede goal-directed behavior in a variety of contexts, although the veracity of this suggestion is unknown, and consequently in need of investigation. The fourth novel mood profile, referred to as the surface profile, is characterized by scores on all mood dimensions being within the 50–56% range. As such, the surface profile approximates the baseline or waterline test norms originally identified by Morgan (1985), suggesting that this profile would be associated with normal functioning across a range of tasks and environments.

Our findings and those derived from subsequent investigations of the six mood profiles identified in the present study may serve to extend and/or refine existing theoretical frameworks related to the mood construct. For example, Lane and Terry's (2000) conceptual model of relationships between mood and performance emphasized the key role played by depressed mood in moderating the effects of anger and tension on performance. A recent study of more than 73,000 participants in an online experiment suggested several ways in which the conceptual model could be extended, for example, by accounting for the effects of mood regulation, suppression and re-appraisal strategies, use of psychological skills, and greater effort (Lane et al., 2017). Our identification of novel mood profiles will help to inform future iterations of the conceptual model.

The finding of sociodemographic differences in the incidence of specific mood profiles clearly warrants further investigation. Males were more likely to report the iceberg profile, whereas females were more likely to report the inverse iceberg and shark fin profiles. Given that the lifetime prevalence of clinical mood disorders in women has been shown to be approximately twice that of men (Steiner et al., 2003), the finding that females were significantly over-represented for the more negative mood profiles is not surprising, and offers support for the predictive validity of the profiles. Sub-clinical negative moods are also more prevalent among females than males (e.g., Butler and Nolen-Hoeksema, 1994; Monteagudo et al., 2013), and gender differences in hormonal activity (Soares, 2013) and use of mood regulation strategies such as rumination (Nolen-Hoeksema and Jackson, 2001) have been proposed as mechanisms to explain the greater prevalence of negative mood profiles among women.

Findings about age-related effects on the incidence of specific mood profiles showed the 25–35 age group to be under-represented for the iceberg profile and over-represented for the inverse Everest and inverse iceberg profile. This trend is consistent with the age-of-onset distribution reported by Kessler et al. (2005), who identified 30 as the median age for mood disorders to emerge, and the lifetime incidence of mood disorders to be 20.8%. Given that the inverse Everest profile was reported by ~3% of the total number of participants in the present study, and the inverse iceberg profile by another 11% of participants, these two negative mood profiles may be indicative of risk of clinical mood disorders and hence could have utility for mental health screening purposes (e.g., van Wijk et al., 2013).

No clear trends emerged for the incidence of specific mood profiles according to level of education. This is consistent with recent overviews of the literature around the influence of education and socioeconomic status on mood disorders, notably bipolar disorder (Schoeyen et al., 2011; Eid et al., 2013). Further investigation of the relationship between education status and mood responses is worthwhile, although other sociodemographic variables appear more likely to yield meaningful insights. The trends for incidence of specific mood profiles by ethnicity were complex and should be treated with caution. There is strong evidence of differences in health status according to ethnicity, including variations in the incidence of mood disorders (Johnson-Lawrence et al., 2013). Given that examination of the link between mood profiles and ethnic background was not the central focus of the present study, we advise against drawing conclusions about the link from our data and recommend that further investigations be conducted in this area.

From an applied practitioner perspective, identification of the six mood profile clusters may assist the interpretation of BRUMS test scores and may extend the utility of the measure in quantifying mood responses by providing a point of reference for attaching meaning to the mood profile. Moreover, the inverse Everest profile may function as an indicator of potential psychopathology among non-clinical samples. Determining the therapeutic meaningfulness and predictive effectiveness of mood profiles appear to be logical directions for future research. Further, the empirical examination of potential links between specific mood profiles and dimensions of personality according to the five-factor model (i.e., extraversion, agreeableness, conscientiousness, neuroticism, and openness to experience; Costa and McCrae, 1992) may also yield beneficial findings, from both theoretical and practical standpoints.

### Strengths and limitations

The primary strength of the current research lies in the fact that the same six mood profile clusters were identified in three large samples that were sociodemographically heterogeneous. The agglomerative, hierarchical cluster analysis followed by the k-means iterative technique produced similar multivariate structures, signaling a robust method of allocation of cases. Although the web-based delivery method and snowballing technique for data collection produced three large and heterogeneous samples, the convenience sampling method may have introduced an element of bias, given that access to the Internet was required for participation. However, replication of the 6-cluster solution in each of the three independent samples represents substantive evidence to support the consistency of the cluster structures.

Limitations are evident regarding the sociodemographic analyses. The small number of participants in the less than high school certificate category (range = 1.7–8.9%) and over 65 age group (range = 0–0.7%) raises the question of whether they adequately represent the underlying populations of interest. Additionally, small cell sizes made results for some analyses uninterpretable and others questionable due to violation of underlying assumptions. Analyses involving the inverse Everest mood profile were most affected given the modest number of participants reporting that profile. Finally, the ecological validity of the mood profiles is still to be determined. However, further investigation of relationships between various aspects of human functioning and the distinct mood profiles identified in the present study seems likely to yield new insights.

## Ethics statement

This study was carried out in accordance with the protocol approved by the University of Southern Queensland's Office of Research and Higher Degrees, Human Research Ethics Committee (approval number: H13REA169). All subjects gave informed consent in accordance with the National Statement on Ethical Conduct in Human Research (2007).

## Author contributions

RP-S—Conception and design of study, acquisition of data, performed analysis on all samples, interpreted data, drafting the manuscript and acted as corresponding author. Final approval of the version to be published. Agreement to be accountable for all aspects of the work in ensuring that questions related to the accuracy or integrity of any part of the work are appropriately investigated and resolved. PT—Conception and design of study, acquisition of data, supervised development of work, helped in data interpretation, drafting and revising the manuscript critically for important intellectual content, final approval of the version to be published. Agreement to be accountable for all aspects of the work in ensuring that questions related to the accuracy or integrity of any part of the work are appropriately investigated and resolved. MM—Conception and design of study, co-supervised development of work, revising the manuscript critically for important intellectual content, final approval of the version to be published. Agreement to be accountable for all aspects of the work in ensuring that questions related to the accuracy or integrity of any part of the work are appropriately investigated and resolved.

### Conflict of interest statement

The authors declare that the research was conducted in the absence of any commercial or financial relationships that could be construed as a potential conflict of interest.
